# About feeding children: factor structure and internal reliability of a survey to assess mealtime strategies and beliefs of early childhood education teachers

**DOI:** 10.1186/s12966-018-0717-x

**Published:** 2018-09-10

**Authors:** Taren Swindle, Madeleine Sigman-Grant, Laurel J. Branen, Janice Fletcher, Susan L. Johnson

**Affiliations:** 1 Department of Family and Preventive Medicine, University of Arkansas for Medical Sciences, 521 Jack Stephens Drive, Little Rock, AR 72205 USA; 2 University of Nevada Cooperative Extension, 2558 So Elizabeth Street, Salt Lake City, UT 85406 USA; 3 Food and Nutrition, University of Idaho, Moscow, ID 83844-3183 USA; 4 Child, Family and Consumer Studies, Center on Disabilities and Human Development, University of Idaho, 1187 Alturas Drive, Moscow, ID 83844-1187 USA; 5 Department of Pediatrics, Anschutz Medical Campus, Section of Nutrition, F-561, University of Colorado–Denver, 12,631 E 17th Ave, Rm #2609, Aurora, CO 80045 USA

**Keywords:** Early care and education, Childcare, Preschoolers, feeding, measurement

## Abstract

**Background:**

Children spend a substantial amount of time in early care and education (ECE) settings and may eat a majority of their diet in this setting. While there are several instruments focused on measuring factors of the ECE environment that may influence diet and weight outcomes, there are few comprehensive, valid, and reliable measures for collecting self-report of ECE providers’ feeding practices. The purpose of this study was to establish the factor structure and internal reliability of a survey developed to measure practices and beliefs of ECE providers relative to feeding children.

**Methods:**

Licensed ECE centers from CA, CO, ID and NV were included in this cross-sectional survey study. The sample was stratified by states and census regions to yield equal numbers of centers from each category. The total sample distribution included 1600 randomly selected centers and up to 8000 staff members (who represented teachers, aides, assistants, or cooks); 1178 surveys were completed. We conducted an exploratory, unrestricted factor analysis as well as parallel analyses to inform the number of factors to be extracted.

**Results:**

Factors within Structural Mealtime Strategies included Adult Control of Foods Consumed (Kuder-Richardson [KR] = 0.67), Bribing with Sweet Foods (KR = 0.70), and Supportive Adult Roles at Mealtime (KR = 0.55). Factors in Verbal Mealtime Strategies included Supporting Children’s Eating Self-regulation (KR =0.61), Pressure to Eat (KR = 0.58), and Social Comparisons (KR = 0.59). Beliefs about Mealtime factors were Autonomy Promoting (α = 0.64), Coercive Beliefs (α = 0.77), and Concern-Based Control (α = 0.60).

**Conclusions:**

The AFC Strategies and Beliefs Survey provides a promising self-report instrument with a strong factor structure consistent with the extant literature to measure practices and beliefs related to feeding and mealtimes in the ECE setting. Feeding young children in group settings differs in many ways from feeding in a family setting; hence it is important that measures such as the AFC Strategies and Beliefs Survey capture unique aspects of the ECE feeding environment.

## Background

According to the National Household Education Survey, 41% of children in the U.S. from birth through five years of age are in nonrelative care outside the home (including those attending center-based care) [1]. Given that children may spend long hours in Early Care and Education (ECE) settings, they may consume up to 2/3 of their daily nutrient intake [2] while away from home. These experiences may influence lifelong eating, which in turn can impact health, including weight status [3]. To address nutritional needs, various “Best Practice” policies have been proposed as suitable standards for use throughout ECE [2, 4]. Best Practice is a term used across health care, business, government, industry, and in our case ECE, to denote “a procedure that has been shown by research and experience to produce optimal results and that is established or proposed as a standard suitable for widespread adoption” [5]. If followed, these guiding documents are proposed to support increased quality of care in the ECE setting. As noted by Larson et al. (2011), not only should ECE settings ensure adequate nutrition, they also should provide “a supportive environment for practicing skills and trying new foods” [6] (p. 1345). Thus, it becomes increasingly important to understand the mealtime environments and feeding strategies ECE providers use to instill healthy eating behaviors in young children using these guidance documents as a framework.

There is a robust literature on parental feeding practices, including both observational and self-reported data, which have identified a number of child feeding constructs. Less work has been done to operationalize child feeding constructs in ECE settings; however, it is likely that some parental feeding constructs have application. Parental feeding strategies that have been predictive of negative child outcomes (e.g., decreased intake of and preference for healthy foods, excess weight, overeating) include authoritarian feeding practices [7–9], restricted access to palatable foods [10–12], use of bribery and coercion [13, 14], permissive and neglectful attitudes towards feeding [15], and pressure to eat [16, 17]. Additionally, perception of the child’s weight [16, 17], cultural influences on child feeding [15, 18], and mealtime structure and routine [19] may modulate adult feeding practices making the study of feeding complex.

Much of the research focused on ECE feeding strategies has relied on constructs and surveys from the parent feeding literature. For example, several studies reporting the impact of ECE mealtime interventions utilized the Child Feeding Questionnaire [20–22] or the Child Feeding Styles Questionnaire [20, 23, 24], both of which were designed for use with parents. While acknowledging that there is terminology in the parental feeding literature that likely applies to ECE feeding, [25] there are also likely to be important differences. Thus, the application of these instruments to educators may not reflect the nuances of unique feeding transactions in this distinct setting or group of caregivers [26]. There are a few studies that have focused on educators specifically. One study by Gubbels and colleagues [27] examined the factor structure and internal consistency of the parent-focused Comprehensive Feeding Practice Questionnaire, as adapted for the ECE providers. Another instrument, the Environmental Policy Assessment and Observation (EPAO) Self Report, includes subscales assessing educator feeding practices and has examined test-retest reliability and validity [28, 29] including predictive value of EPAO-measured provider behaviors on children’s dietary intake. [30] These items focus on whether the teacher sits with the children, what the teacher eats in the classroom, and the teacher’s strategies to encourage healthy eating. Additional development of feeding assessments that are specific to the ECE setting represents an opportunity to capture the complexity of feeding children in group settings and the distinct challenges and teachable moments related to eating and mealtime in which ECE staff participate daily. Such a measure could capture ECE provider feeding practices that have the potential to influence child nutrition and weight outcomes.

Despite the scarcity of tools specific to ECE feeding practices, there are other valuable related assessments. Some existing instruments include items which assess personal nutrition knowledge, dietary intake and weight management behaviors of ECE providers [31–33]; others assess policies and characteristics of the ECE environment related to obesity prevention factors [28, 34, 35]. These instruments have successfully measured environmental factors including food quality, promotion of physical activity, water access, screen time, and nutrition education for the children. Lastly, some self-report measures have focused on menu quality [36] or physical activity [37]. As one specific example, The Wellness Children Care Assessment Tool includes questions about policy related to feeding (e.g., adults not pushing children to eat more). [38]. Thus, while previous instruments have measured some aspects of feeding children in group settings, the development of a tool specifically designed for the group feeding context of ECE, based upon input from ECE directors, staff and policy makers, represents a potential addition to existing methodology.

The About Feeding Children Study was conducted between 2002 and 2005. [39] The original questionnaire was developed, in part, to capture the strategies and beliefs of ECE providers during mealtimes. As noted above, since that time other instruments have been designed to investigate feeding practices in the ECE setting. While incorporating some features of the ECE setting, many constructs still pertain to parental feeding. Given that the AFC study was constructed almost entirely from the ECE perspective, the aim of this study was to explore the psychometrics of the AFC questionnaire (e.g., factor structure, internal reliability), shorten its length, and determine its potential utility for use in research and practice to measure feeding children in group settings.

## Methods

### Study design

The AFC group was a consortium of nutrition and child development professionals from academic institutions in three western states. The consortium focused on enhancing children’s feeding and mealtime experiences in the child care setting. The purpose of the AFC Survey was to expand our knowledge of mealtimes in ECE centers across four Western states—California (CA), Colorado (CO), Idaho (ID), and Nevada (NV). As part of a larger study, a survey targeted to ECE providers from licensed centers serving children 18–60 months of age was designed and conducted. We aimed to capture diversity with respect to geographic areas, center size, ethnicity and level of experience of ECE providers. Our specific aims included gathering data regarding factors influencing ECE providers’ child feeding practices and framing these data in the context of providers’ experience, training, and personal health characteristics. The Institutional Review Boards from each university approved all aspects of this research. Informed consent was obtained from all participants. This included implied consent from those returning surveys and signed consent from those in face-to-face interviews.

### Setting and participants

A stratified random sample of licensed ECE centers from CA, CO, ID and NV was selected, and English-speaking staff from these centers who worked with children 18–60 months of age were invited to participate in the survey. Licensing and accreditation agencies within each state supplied databases of ECE centers, and Head Start and the Child and Adult Care Food (CACFP) Programs from CO, ID and NV also provided information. The final database contained 11,661 centers. Among center-based ECE there are several different types: (1) programs that are federally funded and regulated, and have specific education requirements for staff (e.g., Head Start), (2) programs that are state funded and regulated, and (3) programs that adhere to minimum licensing standards only (e.g., private). The sample was stratified by states and census regions to yield equal numbers of centers from each state and census region. The number sampled per state (*n* = 400) was chosen based on the fewest number of licensed centers within a state (NV with *n* = 418). The total sample distribution included 1600 randomly selected centers and up to 8000 staff members (5 per center who could be teachers, aides, assistants, or cooks). [39]

### Development of questionnaire

#### Initial stage

**Fig. 1:**
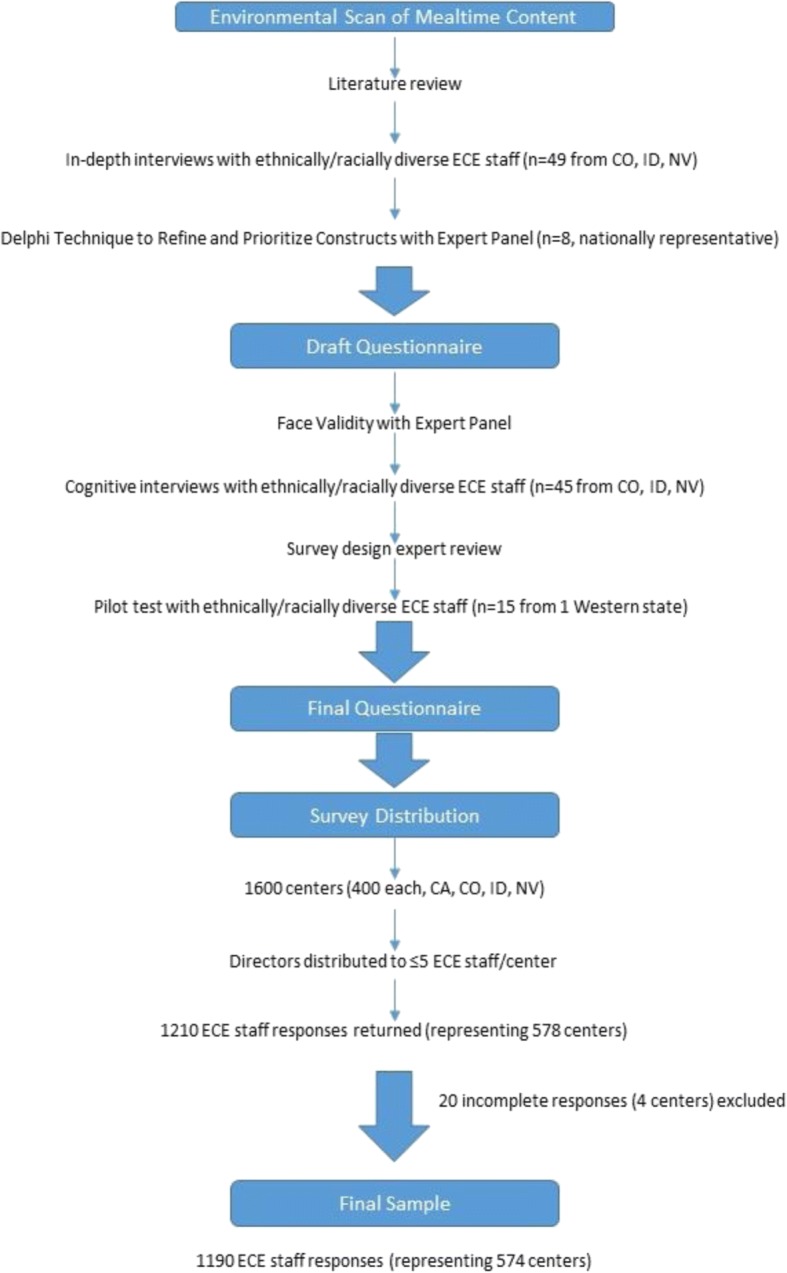
About feeding children strategies and beliefs survey questionnaire development, study design and survey distribution

Figure 1 depicts the development process, including steps to identify content for the AFC survey. In 2001, the research team reviewed the extant literature along with professional guidelines from CACFP, Head Start, American Academy of Pediatrics, National Association for the Education of Young Children, and American Dietetic Association [39]. A list of 13 guidelines was compiled. During the review, it was noted that few instruments regarding the ECE environment from the perspective of the physical, emotional, and social developmental needs of the center, the staff, and the child existed at that time. In 2001–2002, in-depth interviews were conducted in English by trained professionals following a detailed script. Providers were asked to describe ECE ideal feeding experiences; current child-feeding beliefs and practices; interpretation of feeding guidelines; concerns about child feeding; and knowledge of child development and nutrition information. This phase concluded with a stakeholder meeting in 2002 during which national experts participated in operationalizing feeding guidelines and constructs. The expert panel consisted of one parent; two center directors; two feeding sponsors; three CACFP administrators (representing CO, ID, and NV); one specialist for children with special needs; and one licensing authority. Using a modified Delphi method, each member was asked to individually rank their top seven guidelines from a list of 19 generated from existing literature and the aforementioned 13 guidelines. Next, the expert panel was organized into three groups to reprioritize the guidance. Three major themes were generated: allowing children to self-serve, having adults eat the same foods as the children, and not using food as a reward and punishment. The combined information from each study component was used to identify further potential survey themes. Then the entire group reconvened to discuss items to be included in the questionnaire. Following this activity, panel members met in small groups to discuss what behaviors would need to be observed to operationalize each guideline into “Best Practice.” Interviews and meetings were audiotaped and transcribed, and field notes were added.

#### Content of survey

Based upon these data for potential survey themes, we identified constructs related to feeding and crafted items that tapped these constructs. The following topic areas included in the survey: 1) current child feeding practices, 2) the degree of external control exerted in child feeding practices, 3) administrative routines used in child feeding, and 4) perceived barriers to creating optimal mealtimes for children. Participants were directed to consider their usual mealtime experiences only, as it was determined from the interviews that providers view snack times as serving a different purpose than mealtimes. ECE staff also provided demographic information including ethnicity, education level and years of experience. Items were predominately forced choice, and response sets were 5–7-point Likert scales or Yes/No responses. The different response formats were developed based upon item-specific feedback from pilot subjects. Endorsement of items was defined and coded to indicate that an item reflected the characteristics of the respondent (i.e., 0 = never or infrequently; 1 = some to all of the time), consistent with survey research terminology. [39] Although scales differed by item set, this definition was applied consistently to all feeding practices. This standardized the scale of these items to reduce loadings explained by method variance. Practices that require improvement or change relative to Best Practice guidance were identified as ‘Unsupportive Practice.’

In 2004, drafts of the questionnaire were tested using cognitive interviewing [39] with ECE staff from ethnic groups including Hispanic, African American, and Non-Hispanic White. Items and responses were evaluated and adjusted for readability, comprehension, perceptions, completeness of response sets, and clarity of instructions. Items then were revised and reviewed for content and format by a survey design expert. After further refinement, the survey was pilot-tested with 15 individuals in the Denver area who varied in ethnic origin. Researchers noted time to complete the entire survey (mean = 20.2 ± 7.2 min), any missed items, and items for which no response was recorded. Minor changes were made, and the final survey included 60 questions, some of which requested multiple responses, for a total of 171 items. [39]

#### Format

Surveys were formatted using TELEform Elite v8. [39] This automated system allows for scanning of paper-based surveys to extract data directly into datafiles.

#### Distribution of surveys

The tailored design method for unsolicited mailed questionnaires was closely followed. [39] Using this approach, beginning in June 2004, approximately one week prior to mailing, pre-notification letters announcing the survey were sent to state licensing agencies and directors of the 1600 centers. Center director letters included a flyer to post, with the aim of alerting staff about the study.

Packets containing five staff questionnaires, along with an explanatory cover letter and small educational incentives, were sent to ECE directors. Directors were instructed to make materials available to facilitate staff’s ability to anonymously complete the survey. The ECE director determined where and how surveys were distributed but were instructed to maintain staff anonymity. A pre-stamped return envelope was included with each staff survey so that the survey could be anonymously completed, sealed and mailed immediately. One week after the first mailing, a thank you/reminder flyer was sent to directors for posting. Another reminder was distributed two months later. Receipt of returned surveys was logged daily into a database using tracking code labels. Surveys were visually checked for torn and missing pages. Surveys were scanned, verified for errors, and flagged data fields were either corrected or coded as missing. A 41% center response rate was achieved.

#### Data analyses

Participant characteristics including means, SDs, and frequencies were computed. Descriptive statistics and distributions were calculated for each item response of each caregiver survey returned (*N* = 1178, representing 568 centers). Given the broader scope of the AFC survey, 55 of the 171 items were extracted for these analyses. These specific items were selected a priori for inclusion in initial descriptive analyses because they asked about provider child feeding beliefs, attitudes and practices. Excluded item content that was not specific for these analyses included questions about experiences with food insecurity in the classroom, [39] barriers to feeding children (e.g., rules, equipment), and teaching at meals (e.g., manners, social skills). To distinguish this shortened instrument from the original tool, these items will henceforth be referred to as the AFC Strategies and Beliefs Survey.

AFC Strategies and Beliefs Survey items were grouped into three conceptual categories prior to conducting factor analyses: Structural Mealtime Strategies, Verbal Mealtime Strategies, and Beliefs about Mealtime. These categories are consistent with the recent application of the Belsky model [40] to ECE settings, which illustrates how educators’ attitudes and beliefs can predict behaviors (i.e., practices and interactions with children). We further categorized types of behaviors into verbal and non-verbal (i.e., structural). This separation reflects early social modeling literature that documents the differential influence of adults’ words and actions on child behavior. [41–43] Our conceptual grouping would allow for examination of inconsistency between beliefs and practices as well as between actions and words, which may have distinct impacts on children as well. [43] All items were coded such that higher scores were more consistent with supportive practice. Structural and Verbal Mealtime Strategy items with 75% of respondents or greater indicating the same answer were excluded from analyses due to limited variance (i.e., inability to discriminate on the underlying construct) [44]. This is consistent with previous studies measuring mealtime factors, which eliminated items with ceiling or floor effects [45, 46]. There were no ceiling or floor effects observed for the Belief items; therefore, all were retained.

To explore the factor structure of items collected through the AFC Strategies and Beliefs Survey, we conducted an exploratory, unrestricted factor analysis within each construct. Direct oblimin rotation allowed for correlation between items. Analyses were conducted in MPLUS Version 7 [47] using the appropriate correlation matrix for the nature of the variables. That is, Structural and Verbal Strategies were measured or summarized on a binary scale (0,1), and a polychoric correlation matrix was used to appropriately estimate the model with variables of this type. For these models, a weighted least squares estimation (WLSMV) was used to account for the categorical nature of variables [48, 49]. Items in the Beliefs about Mealtime construct were measured on a 1–5 scale. Thus, maximum likelihood estimation was employed for this construct. A parallel analysis [50] was conducted within each conceptual category to determine eigenvalues of factors that would be expected among randomly generated data with no factor structure. In our EFA analyses, only factors with eigenvalues greater than those generated in the parallel analyses were retained. When consistent with previous work done with parents, factors were named to reflect the Content Map of Food Parenting Practices by Vaughn and colleagues. [25] For all constructs, a factor loading greater than .40 was deemed acceptable [48, 49].

Missing data rates ranged from 8.4 to 16.3% per variable in the EFA analysis. Characteristics associated with missing data were identified and included to improve Full Information Maximum Likelihood (FIML) estimation. FIML includes all cases by giving less weight to incomplete response sets and more weight to complete response sets in the final likelihood function [51]. Correlations between factors within constructs were obtained using the FIML estimator. Internal consistency within a factor was estimated using Cronbach’s alpha for scales with continuous items and Kuder-Richardson values for scales composed of binary items. A threshold of .50 was adopted for internal consistency given the limited number of items in suggested sub-scales and the exploratory nature of this study [52, 53].

## Results

Participants with complete data were no different than those with incomplete data in terms of ethnicity, job description (i.e., assistant versus lead educator), gender, employed by Head Start (yes/no), socioeconomic status of families served, geographic area, state surveyed, level, or self-reported BMI. However, participants with missing data were significantly less likely to be from a center with funding from CACFP (*p* = 0.03) and were significantly older (*p* < 0.001) with significantly more years of experience in the field (*p* = 0.001). Thus, these variables were included in model datasets as mechanisms to account for missing data.

### Item descriptive statistics

Table 1 presents sample demographics. Table 2 presents the percent of participants that endorsed (indicating support, approval and/or usage) each item for both Structural and Verbal Mealtime Strategies. Endorsement percentages indicated that eight Mealtime Structure items reflected floor (*n* = 3) and ceiling effects (*n* = 5); these items were not included in further analyses. In total, nine items were excluded from further analyses for Verbal Mealtime Strategies due to limited variability; mostly (*n* = 7) due to ceiling effects. The two items receiving the lowest endorsement both began with “I never.” All Belief items were retained as no ceiling effects were observed. Thus, in summary, 14 items were included for EFA analyses within structural mealtime strategies; 13 items were included for verbal mealtime strategies; and 11 items were included in the EFA for beliefs about mealtime.

**Table 1:** Demographic and personal descriptive statistics for the survey respondents

Provider characteristics	(*N* = 1178)	
	Frequency	
Sex	*n*	*%*
Female	1157	98.2
Male	21	1.7
Race & Ethnicity	*n*	*%*
White	871	73.9
Black	68	5.8
Hispanic (may be White, Black or Other)	179	15.2
Other	225	19.1
Education	n	*%*
High school or less	261	22.2
Some College	435	36.9
Associates Degree	195	16.5
BA/BS Degree	210	17.8
Graduate Degree	56	4.8
Missing	21	1.8
Role at Agency	*N*	%
Lead Teacher	948	80.5
Assistant Teacher	164	13.9
Missing	66	5.6
Center Type (multiple answers may apply)	N	%
Head Start	97	8.2
CACFP	322	27.3
	Mean	SD
Age	36.8	12.7
Body Mass Index (BMI; kg/m^2^)	26.6	6.3
Experience in child care in years	9.5	7.7

**Table 2:** Item pool and endorsement for structural and verbal mealtime strategies

	% Endorsement
*Structural Mealtime Structures*	
When children are very thin, I serve them more of everything.^a^	10.5
To help children eat, I start feeding them.^a^	17.1
If picky children don’t want to eat, I start feeding them so they get interested.^a^	21.2
I don’t let them have seconds of other foods until they try the new food.	25.5
*Both children and staff pass the food.*	31.3
To help children eat, I serve sweet food after they eat the rest of the food on their plates.	35.7
If picky children don’t want to eat, I wait to serve them sweet foods if they do not eat something from their plate.	40.2
The children have to take at least one bite.	42.5
I have the children try the food before they can have sweet foods.	43.9
*I sit with the children at mealtime.*	48.1
*When children are served new food, I try the new food all of the time.*	53.3
*I eat the same food as children at mealtime.*	53.6
I have the children eat one bite of each food.	53.8
I have the children finish their meal before eating sweet foods.	54.8
When children are very thin, I offer them more of the foods they like.	56.4
If children do not want to stop eating, I try to distract them with another activity.	61.7
*Children are involved in serving themselves at mealtime.*	65.1
I have the children eat nutritious food before “junk” food.^a^	82.3
*I let children decide how much to eat.*^a^	83.3
*I try the new food with the children.*^a^	86.4
*I offer new foods at mealtime or snack time.*^a^	87.7
If children do not want to stop eating, I send them away from the table.^a^	91.9
Verbal Mealtime Strategies	
I never ask children if they want more to eat.^a^	5.4
I never encourage children to eat the amount I think they need.^a^	14.0
To help children eat, I point out other children who are eating more.	31.4
If children do not want to stop eating, I ask if their tummy is full.	33.8
I tell the children if they have not eaten enough.	39.8
If picky children don’t want to eat, I let them know they don’t have to eat.	49.3
I say something like “Pat is eating green beans. Why don’t you eat some?”	50.2
If children do not want to stop eating, I explain that they need to leave enough for everyone	50.7
If picky children don’t want to eat, I tell them to take at least one bite of everything.	55.9
When children are very thin, I praise them for eating to get them to eat more.	56.4
To help children eat, I suggest that they need to eat more.	57.9
If picky children don’t want to eat, I ask if their tummy is full.	58.9
If picky children don’t want to eat, I suggest that they start eating what is on their plate.	64.8
If children do not want to stop eating, I tell them they can’t have more of some foods.	67.2
*I ask children if their stomach are full.*	69.7
*I talk about food at mealtime.*^a^	82.0
I notice and comment to the child who is eating well.^a^	84.7
*I teach the children about new foods.*^a^	85.3
If children do not want to stop eating, I tell them they can’t have any more to eat.^a^	87.2
If picky children don’t want to eat, I ask them to eat something on their plate.^a^	87.4
I ask the children to take a bite.^a^	90.5
To help children eat, I explain to the children that the food will make them grow and be healthy.^a^	96.1

^a^Items dropped from further analyses because of limited variability defined as > 75% selecting one response option. Italics are used to indicate items conceived as best practice

### Exploratory factor analysis by construct

#### Structural mealtime strategies

Parallel analysis suggested extraction of factors with eigenvalues greater than 1.2. Three factors met this criterion. These factors were defined as Adult Control of Foods Consumed (e.g., “The children have to take at least one bite.”); Bribing with Sweet Foods (e.g., “I have the children try the food before they can have sweet foods.”); and Supportive Adult Roles at Mealtime (e.g., “I eat the same food as children at mealtime.”). Two items did not load onto any factor (see Table 3). Kuder-Richardson values (indicating internal consistencies) were 0.67, 0.70, and 0.55 for the three factors, respectively. The correlation between Adult Control of Foods Consumed and Bribing with Sweet Foods was significant and positive (*r* = 0.55, *p* < 0.05). Supportive Adult Roles at Mealtime were not significantly correlated with Bribing with Sweet Foods (*r* = 0.37, *p* > 0.05) or Coercive Control (*r* = 0.04, *p* > 0.05).

**Table 3:** Factor loadings and frequency of endorsement for structural mealtime strategies

Item content	Adult control of foods consumed	Bribing with sweet foods	Supportive adult roles at mealtime
I don’t let them have seconds of other foods until they try the new food.	0.74		
The children have to take at least one bite.	0.88		
I have the children eat one bite of each food.	0.76		
I have the children try the food before they can have sweet foods.		0.50	
I have the children finish their meal before eating sweet foods.		0.63	
I wait to serve them sweet foods if they do not eat something from their plate.		0.65	
I serve sweet food after they eat the rest of the food on their plates.		0.98	
I eat the same food as children at mealtime.			0.76
I sit with the children at mealtime.			0.48
Both children and staff pass the food.			0.76
I try new foods with children (all or most of the time).			0.48
I offer them more of the foods they like.	−0.02	0.38	−0.22
I try to distract them with another activity.	0.06	0.28	−0.11

#### Verbal mealtime strategies

Three factors with eigenvalues greater than 1.15 were extracted based on the parallel analysis. Factors 1, 2, and 3 were named Supporting Children’s Eating Self-regulation (e.g., “I ask the children if their stomachs are full.”); Pressure to Eat (e.g., “I tell the children if they have not eaten enough.”); and Social Comparisons (e.g., “I point out other children who are eating more.” Kuder-Richardson values were 0.61, 0.58, and 0.59 respectively. Three items did not load onto any factor and were eliminated (Table 4). The Supporting Children’s Eating Self-regulation and Social Comparisons factors were negatively correlated (*r* = − 0.31, *p* < 0.05). Pressure to Eat was not correlated to either Supporting Children’s Eating Self-regulation (*r* = − 0.18, *p* > 0.05) or Social Comparisons (*r* = 0.39, *p* > 0.05).

**Table 4:** Factor loadings verbal mealtime strategies

Item content	Autonomy supporting cues	Autonomy undermining cues	Social comparisons
I ask the children if their stomachs are full.	0.76		
If children do not want to stop eating, I ask if their tummy is full.	0.53		
If picky children don’t want to eat, I ask if their tummy is full.	0.88		
I tell the children if they have not eaten enough.		0.78	
If picky children don’t want to eat, I suggest that they start eating what is on their plate.		0.46	
If picky children don’t want to eat, I let them know they don’t have to eat.		0.51	
To help children eat, I suggest that they need to eat more.		0.63	
I say something like “Pat is eating green beans. Why don’t you eat some?”			0.80
I point out other children who are eating more			0.86
If children do not want to stop eating, I tell them they can’t have more of some foods.	−0.04	0.25	0.24
If picky children don’t want to eat, I tell them to take at least one bite of everything.	−0.03	0.34	0.28
When children are very thin, I praise them for eating to get them to eat more.	−0.01	0.36	0.36

#### Beliefs about mealtime

**Fig. 2:**
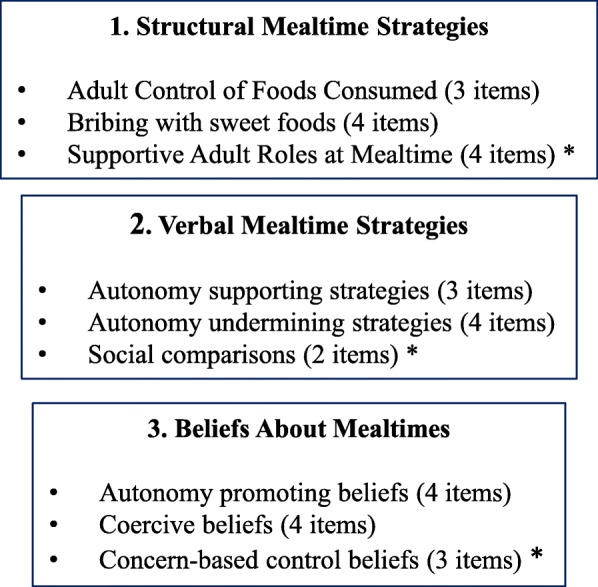
Components and constructs of the about feeding children mealtime strategies and beliefs survey

Similar to those for the other constructs, a parallel analysis suggested extraction of three factors for Beliefs about Mealtime (eigenvalues > 1.15). The first factor was designated as Autonomy Promoting Beliefs (Cronbach’s alpha = 0.64; e.g., “Trying the new food with them will work to get children to try new foods.”). The second factor was designated as Coercive Beliefs (Cronbach’s alpha = 0.77; e.g., “Having a “one bite” rule will work to get children to try new foods.”). The third factor was designated as Concern-Based Control Beliefs (Cronbach’s alpha = 0.60; “When a child is feeling sad, it’s okay to offer a cracker to help the child feel better.”) (see Table 5). Autonomy Promoting Beliefs was not correlated with Coercive Beliefs (*r* = − 0.03, *p* > 0.05) or with Concern-Based Control (*r* = 0.01, *p* > 0.05). Coercive Beliefs and Concern-Based were positively, significantly correlated (*r* = 0.30, *p* < 0.05). Figure 2 presents all components and constructs.

**Table 5:** Factor loadings beliefs about mealtime

Item content	Autonomy promoting beliefs	Coercive beliefs	Concern-based control	Mean (sd)
Teaching children about new foods before offering the foods at mealtime will work to get children to try new foods.	0.66			2.61 (0.67)
Trying the new food with them will work to get children to try new foods.	0.81			2.37 (0.70)
Children are more likely to try a new food after they see me eat it.	0.43			1.99 (0.84)
Having the new food on the table at mealtime and letting children decide when to try it will work to get children to try new foods.	0.42			2.72 (0.90)
Having a “one bite” rule will work to get children to try new foods.		0.63		2.72 (0.93)
Keeping them from having sweet foods until they try the new food will work to get children to try new foods.		0.72		2.88 (0.99)
Not having seconds of other foods unless they try the new food		0.83		3.08 (1.01)
Suggesting they try a bite		−0.43		2.56 (0.74)
Adults know better than children how much children need to eat.			.81	3.48 (1.25)
If children put food on their plates, they should eat it.			.45	3.37 (1.27)
When a child is feeling sad, it’s okay to offer a cracker to help the child feel better.			.41	3.76 (1.20)

All belief items were measursed on 1–5 scale with lower scores corresponding to greater agreement. No items were reverse-coded before mean estimation

## Discussion

The AFC Strategies and Beliefs Survey was designed to assess feeding beliefs and practices of ECE providers during mealtimes in ECE centers. The factor analyses and endorsement patterns suggest that the AFC Strategies and Beliefs Survey captured both Best Practice Guidance and Unsupportive Practice. Comparison of the AFC survey items with the American Academy of Nutrition and Dietetics benchmarks for nutrition in child care [20, 54] reveals that of the 12 guidance categories outlined by this position paper, 5 are addressed by the AFC survey (providers sit with children, providers eat meals with children, meals are served “family style,” providers help children recognize internal cues, providers do not use controlling feeding practices). Two additional benchmarks (providers model healthful eating and providers teach children about nutrition) were included as items in the original survey but, due to > 75% endorsement of these strategies (and therefore limited variance or discrimination added by these items), were dropped for factor analyses. Benchmarks that were not addressed included information about training and education for providers, children and parents; these were gathered by our larger survey but not included in this analysis of feeding practices and beliefs. A further strength of the AFC Strategies and Beliefs Survey is that it includes items related to beliefs underlying the benchmarks that were assessed (e.g., beliefs about teaching children, attitudes about what works at meals, beliefs about children’s self-regulation).

Results from exploratory factor analysis, parallel analysis, internal consistency estimates, and factor inter-correlations converge to suggest that the factors presented are definite, distinct, and internally reliable. This instrument is complementary to, yet distinct from, existing measures focusing on elements of ECE policies and environmental characteristics [55–57]. The inclusion of both beliefs and practices in the AFC survey provides a potential opportunity to document ECE self-reported strategies at mealtime but also to determine whether beliefs about child-feeding align with reported behaviors. Previously, only one instrument has included items about beliefs, knowledge and practices but not necessarily with an alignment among these items that could facilitate comparisons [58]. Additionally, based upon stakeholder input, items were developed which specifically asked about difficult to manage transactions—how to manage the presence of highly palatable, sweet foods that children seek to consume. These items provided insights into linkage of beliefs and practices. While not all ECE staff endorsed the belief that withholding sweet foods helps to get children to try new foods (Mean, SD = 2.88, 0.99), > 40% of participants responded that they used this strategy to encourage children to try new foods. Additionally, the inclusion of items which capture verbal as well as structural practices is a strength and is unique to the AFC survey; verbal influences, such as praise and social comparisons, may be important in influencing child behaviors—negatively or positively and perhaps differentially over time.

It should be acknowledged that the AFC survey focuses on *feeding* beliefs and strategies and does not address some important constructs measured by other instruments. For example, other instruments capture environmental influences on mealtime quality (e.g., noise level), [28, 55] the food environment [28], the use of nutrition education techniques [28], child involvement in food preparation, [27] screen use during mealtimes, [24, 28, 55] and policies related to mealtimes in childcare [28]. That said, one advantage of the AFC survey is that its narrower focus allows for multiple items to address a single construct, which should produce a more robust assessment of practices and beliefs [44].

The development of the AFC Strategies and Beliefs survey was undertaken in a manner to reflect practices unique to a communal feeding setting with elements that may impact those practices. While there are feeding practices utilized by ECE staff that are consistent with parenting practices in the home setting, there also are unique practices that are necessitated by feeding children in group settings. Examples of such practices that were included in the AFC survey include social comparisons regarding children’s eating behaviors (which can be easily made, given the number of children participating in mealtimes) and an emphasis on staff eating with children and engaging with children to pass food around the table. While a number of constructs related to parental feeding practices were not included in this survey (e.g., monitoring children’s intake, perceived responsibility in child feeding [9]), these constructs do appear to some extent in other instruments that have been recently developed for the ECE setting [24, 27, 28, 55].

The resulting survey instrument provides a tool that measures nine distinct factors within three ECE child-feeding constructs (Fig. 2). Of the nine factors, six are consistent with the Vaughn et al. Content Map of Food Parenting Practices. [25] Unique factors were Social Comparisons, Concern-Based Control Beliefs, and Supportive Adult Roles at Mealtime which capture practices across two Vaughn Structures (Meal and Snack Routines and Modeling) and is consistent with other terminology in the literature. [59] Social Comparisons, while possible in a home environment, may be a strategy that is more likely to be used in the ECE feeding environment. Regardless, this is a potential difference with the conceptual model that has been constructed for parental feeding practices. Finally, Concern-Based Control Beliefs reflected things adults believe they should do for the child’s best interest. Educators may have concerns about children’s food insecurity, [40] feel accountable for ensuring children eat enough, and generally want children to feel positive in their classroom. This feeling of responsibility may result in a variety of thoughts and actions. Further work to determine the overlap and differences of feeding practices for parents and ECE is needed and could result in parallel maps that align with both home and child care environments.

Concerning the findings more broadly, as expected, ECE educators simultaneously agreed with practices and held beliefs that were both supportive and unsupportive of children’s healthy eating development. To add further to the complexity of the ECE environment, educators indicated use of Best Practice Guidance without believing in their effectiveness for feeding young children. To fully represent and understand the impact of educators’ feeding practices, we suggest all aspects should be included in measures of the ECE feeding environment.

### Structural mealtime strategies

Adult Control of Foods Consumed, Bribing with Sweet Foods, and Supportive Adult Roles at Mealtime each were distinct and strong factors within Structural Mealtime Strategies. The Adult Control of Foods Consumed factor reflected items focused on ensuring children try all foods served (including new foods). While some have advocated for these strategies (e.g., Two Bite Club [60]), these practices may ignore child hunger/satiety and fail to value child choice [61]. The long-term impact of one or two bite “rules,” rewards associated with trying foods, and the level of forcefulness with which these are employed, have been reported to have varying relationships with children’s outcomes in a trial involving parents and in an experimental study without parents [62, 63].

The Bribing with Sweet Foods factor included items reflective of teacher strategies to withhold sweet foods as an incentive until children ate other foods served [14, 63]. Using ECE educators’ input during development of these items, “sweet foods” was a term adopted broadly to include dessert foods *and fruit*. Through formative research to develop the items, we gleaned that this term reflected educators’ position that fruit was a highly desirable food for children and could be considered to elicit as many problematic behaviors at mealtime as dessert. Existing literature (experimental, observational with parents, and intervention testing in schools) suggests that controlling access to food in this way is counterproductive and does not support intake of, or preference for “healthy” foods [13, 14, 64–66].

Supportive Adult Roles at Mealtime reflected items conceived as Best Practice Guidance (i.e., ECE staff sits, eats, and tries new food with children; children and staff pass foods). These practices are consistent with family-style meal service, a standard in Head Start settings [67] and an evidence-based guideline issued by the Academy of Nutrition and Dietetics. [68] Educators sitting with children and eating the same food has been associated with increased vegetable intake for children [69, 70] but has also been reported to be difficult to achieve, particularly when staffing is inadequate or when environmental demands are too high [71]. Conceptually, such strategies reflect previously observed parental modeling constructs [72, 73] that might be applicable to the ECE setting.

### Verbal mealtime strategies

Supporting Children’s Eating Self-regulation, Pressure to Eat, and Social Comparisons emerged as Verbal Strategies. Items in Supporting Children’s Eating Self-regulation focused on directing children to their own sense of fullness and hunger to guide their eating. Use of Supporting Children’s Eating Self-regulation may promote long-term self-regulation of eating and support children’s attending to their internal signals of hunger and satiety, whereas adult directives can override or ignore the internal state of the child [54, 74–77]. The Pressure to Eat factor was comprised of items measuring educators’ use of comments to guide children’s eating based on their own perceptions (as opposed to the child’s hunger/satiety) of how much the child needs to eat. Use of external adult control in the parental feeding domain has been associated with problematic outcomes for children’s eating and weight status [9, 17, 78]. The third factor in Verbal Strategies, Social Comparisons, was comprised of two items that were less often endorsed, relative to other items. The content of these items reflected strategies to compare the amount or types of foods (e.g., green beans) eaten between a target child and another child in the classroom. Research on the impact of social comparisons on classroom learning has been found to lead to decreases in child motivation and empowerment [79], though less work has been reported on the use of social comparisons and its impacts on children’s dietary intake and feeding.

### Beliefs about mealtimes

Autonomy Promoting items reflected 1) teaching about foods (e.g. source, sensory properties and nutritional value) to create a positive atmosphere for children to learn about and try new foods and 2) role modeling to encourage children to eat fruits and vegetables [72]. This is an important construct given the previously documented relationship between beliefs and nutrition education and parenting communication efforts of ECE staff [80]. Coercive Beliefs reflected items that queried the effectiveness of pressuring children to try new foods (e.g., withholding seconds of other foods, the ‘one bite’ rule). Despite the detrimental impact of behaviors such as instrumental use of foods on child preference [13], mean scores for these items were near 3 (out of 5), reflecting variability or indecision regarding how to approach the introduction of novel foods to young children.

Concern-Based Control Beliefs, the final factor to emerge, reflected beliefs that adults know better than children how much, what and when food should be consumed. These items displayed the greatest mean scores of all the Belief items, suggesting that like parents, ECE educators desire to see children eat and feel that controlling mealtimes to that end is part of their responsibility [81]. When children do not eat the desired amount or types of foods, educators may feel it is in the best interest of the child for the adult to intervene.

### Endorsement of strategies

ECE staff endorsed items consistent with Best Practice Guidance at varying rates, whereas items *inconsistent* with Best Practice Guidance most often received majority endorsement (i.e., staff endorsed practices that are not considered Best Practice). Consideration of the level of endorsement of all items, regardless of their retention based on variability in the factor analyses, provides a useful reflection on the ECE feeding environment. For example, within Structural Mealtime Strategies, the majority of educators (> 75%) endorsed supportive practices such as offering new foods, trying new foods, and letting children decide how much to eat. On the other hand, the majority also endorsed Unsupportive Practices, such as sending children away from the table when they do not want to stop eating and controlling the order in which children consumed foods. The challenges of helping children learn to recognize internal cues of satiety (to terminate eating), even when palatable food is still available, has not been studied, despite reports that ECE staff struggle with this situation, particularly when children come from food insecure households [82–84].

Similarly, within Verbal Mealtime Strategies, > 75% endorsed supportive practices such as talking about all foods and teaching about new foods but also sanctioned several items focused on Unsupportive Practices such as getting children to eat more (e.g., comment to the child eating well, ask them to eat something on their plate, ask children to take a bite). Conversely, some items regarded as Best Practice Guidance were endorsed by as few as 31% of educators (e.g., staff and children pass the food). In terms of Beliefs, mean scores consistent for Unsupportive Practices (e.g., adults know better than a child how much to eat) were higher than those aligned with supportive practice (e.g., children will try if they see me eat it). Overall, these findings suggest that there may be more room for training and intervention to increase adoption of best practices and to de-implement (i.e., remove, replace, reduce) [85, 86] practices and change beliefs that are not supportive of children’s development.

### Limitations and strengths

Our systematic approach to the development of this instrument has both limitations and strengths. A limitation to the potential generalizability of this work is the focus on data collection in Western states in the US in center-based care. Our results may not reflect the practices and beliefs of educators in other parts of the US or home-based providers. Another limitation is that three of the factors exhibited lower levels of internal consistency (> 0.50 and < 0.60) as indicated by Kuder-Richardson values [44]. The goal of this study was to develop a tool with the least number of items that measure distinct constructs, with as little redundancy as possible. Though brevity is known to lead to lower internal consistency values compared to instruments with more items and greater redundancy [62, 63], given the objective of this study, pragmatic and reliability concerns were balanced. Finally, the wording of some items should be considered in relation to endorsement and conception of Best Practice Guidance. For example, strong, unambiguous wording is used in survey item development to avoid acquiescence bias and increase variability on the latent construct [44]. In this study where a binary response option was coded for Structural and Mealtime Strategies items, extreme wording was related to very low endorsement (< 15%) for items using words like “very” and “never.” Moreover, the inconsistency in terms of degree of endorsement between agreement with practice and belief in practice suggest either a true disconnect between practice and belief, fear of reprisals, or social bias in reporting. Further development of the tool could assess the psychometric properties of the Feeding Strategies scales using consistent and full Likert scales and estimate intraclass correlation coefficients between centers in the same center and agency. Exploration of feeding practices at snack in child care settings is also warranted.

A primary strength of the AFC Strategies and Beliefs Survey was its employment of qualitative methods (expert input, survey design consultation, and cognitive testing interviews) with diverse populations and incorporation of feedback at each level of the survey design. This process was utilized to ensure that response sets were complete and that providers interpreted the questions and responses in a similar manner. Further, 78% of educators in our sample had at least some college education, and 23% had achieved either an undergraduate or graduate level degree. This level of education is slightly lower than national estimates that report 53% of ECE settings-based and 30% of home-based providers have a college degree [87]. Finally, the inclusion of items within each construct of the AFC Strategies and Beliefs Survey that reflect both Best Practices and those strategies which fall short of these practices provides the ability to capture the range of classroom practices and beliefs related to feeding in ECE.

## Conclusions

The AFC Strategies and Beliefs Survey is a promising self-report instrument with a strong factor structure consistent with the extant literature to measure practices and beliefs related to feeding and mealtimes specifically in the ECE setting. Feeding young children in group settings differs in many ways from feeding in a family setting; hence it is important that measures such as the AFC Strategies and Beliefs Survey capture the unique aspects of the ECE feeding environment. Further, this research adds to the growing body of areas of strength and opportunity, particularly those investigating cutting edge strategies for de-implementation of detrimental (i.e., unsupportive) beliefs and practices. Additional development work with the AFC Strategies and Beliefs instrument should explore test-retest reliability, convergent validity with other measures in the field, and predictive validity of this tool against classroom observations and child outcomes. Future studies also could look at group differences on these constructs among educators with varying characteristics (e.g., education, training, experience, ethnicity/culture, age of children served, population served, demands of food insecurity, provider personal characteristics like weight status and eating history). Finally, the sensitivity of this instrument in response to intervention should be explored. ECE educators are responsible for employing Best Practice Guidance in every aspect related to child development, including the feeding environment.

## Abbreviations

AFC: about feeding children
BMI: Body mass index
CA: California
CACFP: Child and adult care food program
CO: Colorado
ECE: early care and education
EFA: exploratory factor analysis
FIML: Full Information Maximum Likelihood
ID: Idaho
KR: Kuder-Richardson
NV: Nevada
SD: Standard deviation
US: United States
WLSMV: weighted least squares estimation
